# Chronic Exertional Compartment Syndrome of Bilateral Lower Limbs and Forearms in an Elite Ice Hockey Athlete: A Case Report

**DOI:** 10.7759/cureus.84483

**Published:** 2025-05-20

**Authors:** Kevin Y Zhao, Peter Staunton, Simon Martel, Louis-Nicolas Veilleux, Drew Schupbach, Thierry Pauyo

**Affiliations:** 1 Division of Orthopaedic Surgery, McGill University, Montreal, CAN; 2 Motion Analysis Center, Shriners Hospitals for Children - Canada, Montreal, CAN

**Keywords:** athlete, cecs, chronic exertional compartment syndrome, ice hockey, intracompartmental pressure monitor

## Abstract

Chronic exertional compartment syndrome (CECS) involves increased intracompartmental pressures (ICPs) induced by physical activity, leading to intense pain and associated neurological symptoms that resolve with rest. Classically, it is associated with young male athletes. The lower limbs are typically affected in running athletes and marching military members, while the upper limbs are generally involved in motorcyclists and rowers. Conservative treatment options involve activity modification, such as alteration of foot strike patterns and botulinum injections, while surgical treatments range from open to percutaneous fasciotomy. CECS is rare and remains a challenging diagnosis. In addition to history and physical exam, magnetic resonance imaging and intracompartmental measurements throughout exercise stress tests are described. In this article, we outline the first reported case of CECS in an ice hockey athlete involving all four limbs that was successfully diagnosed with a continuous ICP monitor and treated with open fasciotomy of all four limbs.

## Introduction

Chronic exertional compartment syndrome (CECS) is a rare and underdiagnosed condition that predominantly affects young male athletes [1]. It causes symptoms of progressive pain, tightness, or numbness during exercise activity that resolve following activity cessation [2]. The exact pathophysiology of CECS remains debated. However, it is commonly understood to involve pathologically elevated compartmental pressures caused by various factors, including fascial thickening or elasticity, microtrauma to muscle, myopathies, and vascular compromise [3].

CECS is most classically seen in the lower limbs, with a reported incidence rate of 33% in active patients presenting with exercise-induced leg pain [4]. The currently accepted diagnostic criteria for CECS remain controversial. These criteria were proposed by Pedowitz et al. in 1990 and rely on static intracompartmental pressure (ICP) measurements made at single time points before and after exercise [5]. Diagnostic criteria for CECS in the presence of appropriate clinical symptoms include 1) a resting compartment pressure ≥ 15 mmHg, 2) a pressure of ≥ 30 mmHg one minute after exercise, or 3) a pressure ≥ 20 mmHg five minutes after exercise [5]. However, these diagnostic criteria were established for lower limb CECS, as cases involving the forearms are a much rarer entity with an unclear incidence rate and vaguely defined diagnostic criteria [6].

CECS of the lower limbs tends to present in running athletes and marching military members [7,8], while upper limb CECS is most commonly seen in motorcyclists and rowers [9,10]. To our knowledge, there has been no previous report of an individual diagnosed with CECS of both lower limbs and forearms or in an ice hockey athlete in the literature. In this report, we present a case of CECS involving bilateral lower limbs and forearms in an elite male ice hockey athlete and outline the diagnostic and treatment plans.

## Case presentation

A 17-year-old male competitive ice hockey athlete presented with complaints of recurrent lower limb pain for the past six months. Both limbs were affected equally, and the pain was mainly localized to the calves. The pain was associated with numbness anterolaterally and posteriorly. The symptoms consistently occurred when pushing off while skating in ice hockey and would gradually subside with rest, resulting in complete symptom resolution after 30 seconds. The patient reported no previous medical history and no history of trauma to either lower limb. 

**Figure 1:**
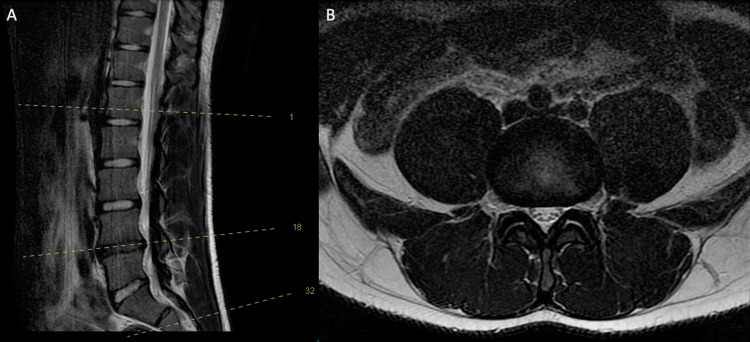
Magnetic resonance imaging (MRI) of the lumbar spine A: Sagittal view with reference line; B: Axial view at the L4-L5 level

On examination of the lower limbs, there was no evidence of swelling, erythema, or deformity. Motor function, sensation, and tone in both lower extremities were normal. Dorsalis pedis and posterior tibial artery pulses were palpable. The symptoms were reproduced after jumping on one leg for 30 seconds and gradually improved with complete resolution two minutes after sitting down. Plain radiographs of both lower limbs were normal, with no evidence of fracture or medial tibial stress syndrome. Magnetic resonance imaging (MRI) of the lumbar spine showed degenerative disc disease at the L4-L5 level with disc space narrowing and diffuse disc bulging indenting both L4 nerve roots. There was no significant compression and no evidence of central stenosis (Figure 1). This was not pursued further as a cause of the patient’s symptoms, as there was no suggestion of radiculopathy on history or physical exam.

**Figure 2:**
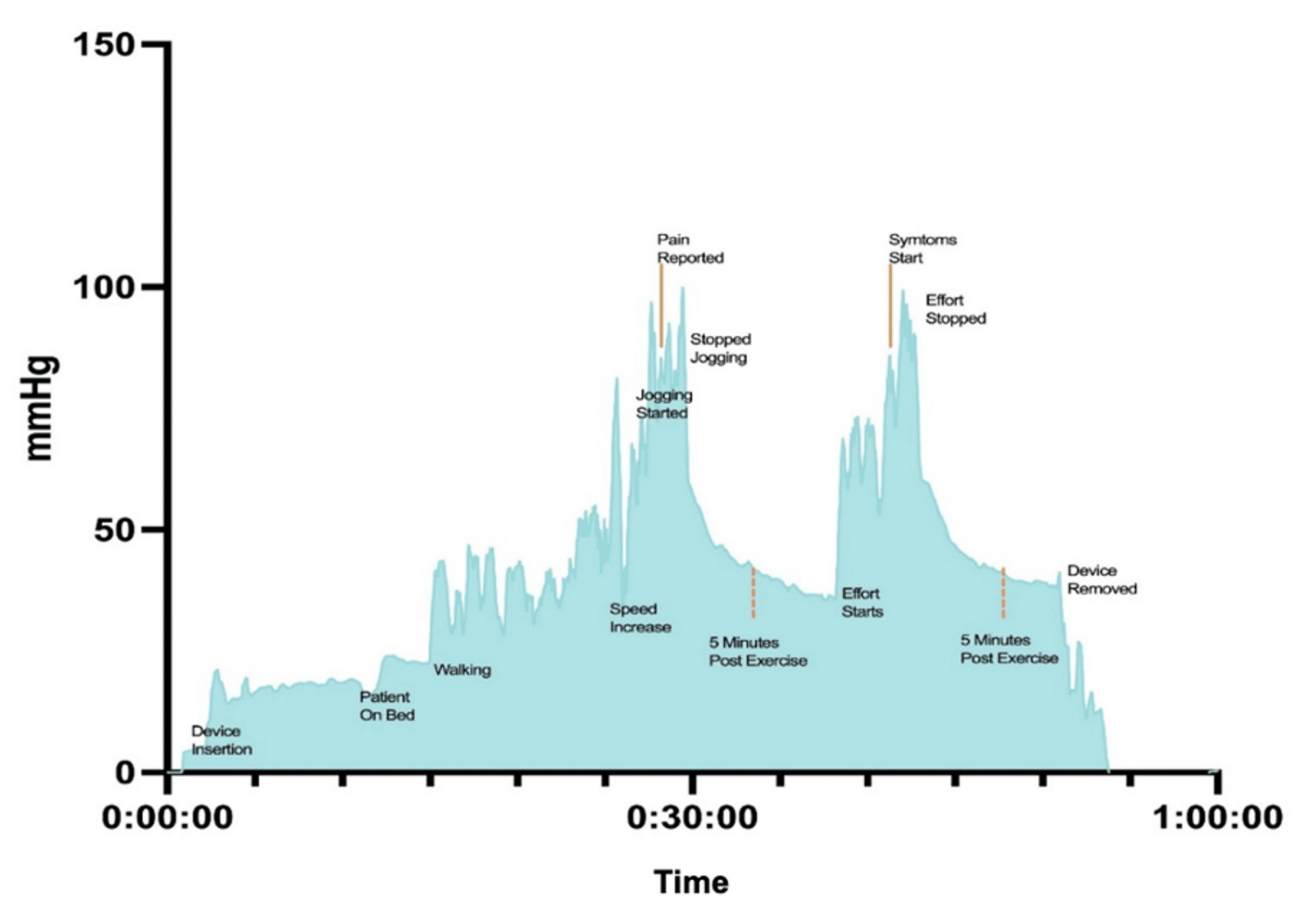
Intracompartmental pressure curve of the right posterior compartment during a treadmill exercise challenge

Continuous ICP monitoring of the right posterior compartment was performed using a digital device (MY01, Montreal, QC, Canada) [11,12] while the patient ran on a treadmill until symptoms were reproduced. The ICP was 19 mmHg prior to initiating exercise. At peak effort, the ICP increased up to 108 mmHg. At five minutes post exercise, the ICP remained elevated at 40 mmHg (Figure 2). Symptoms gradually improved following cessation of the treadmill activity. The ICP measurements met all three diagnostic criteria, of which only one is required, for lower limb CECS outlined by Pedowitz et al. (Table 1) [5]. The patient was diagnosed with chronic exertional compartment syndrome of the lower limbs bilaterally, and surgical treatment with fasciotomy was offered following a discussion regarding the potential benefits and risks. Accordingly, the patient underwent bilateral open four-compartment fasciotomy of the anterior, lateral, superficial posterior, and deep posterior compartments under tourniquet control. There were no surgical complications. 

**Table 1:** Continuous intracompartmental pressure measurements at specific time points Based on the chronic exertional compartment syndrome (CECS) diagnostic criteria outlined by Pedowitz et al. for lower limbs for reference [5].

Time point	Lower limbs	Forearms	Pedowitz et al.'s criteria*
At rest	19 mmHg	10 mmHg	≥ 15 mmHg
Peak exercise	108 mmHg	90 mmHg	-
One minute post exercise	50 mmHg	12 mmHg	≥ 30 mmHg
Five minutes post exercise	40 mmHg	-	≥ 20 mmHg
*Originally established for lower limb CECS			

**Figure 3:**
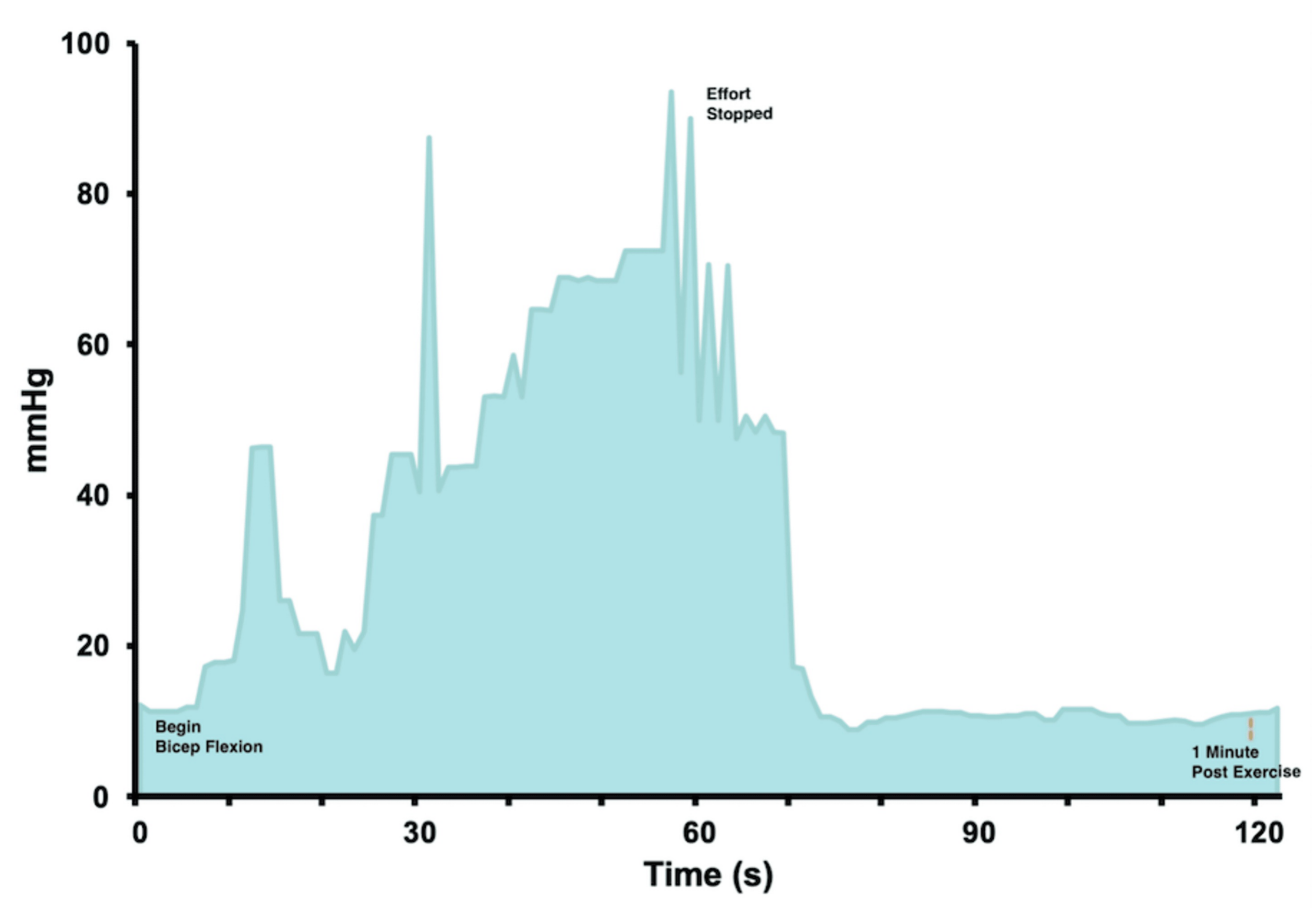
Intracompartmental pressure curve of the forearm during a one-minute biceps flexion exercise challenge

At six months follow-up, the patient resumed competitive hockey with no pain or symptom recurrence in the lower limbs. However, the patient reported pain and paresthesia in the bilateral forearms during physical activity. The pain was non-specifically localized in the forearms, would occur with tight gripping of the hockey stick or with forceful steering while driving, and would gradually improve with rest. Following a trial of activity modification, the patient continued to complain of these symptoms at further interval follow-up. On examination, assessments of range of motion, strength, and sensation were normal in both upper limbs. Electromyography showed normal motor and sensory conduction of the median and ulnar nerves bilaterally. MRI of the cervical spine and both brachial plexuses was normal. The continuous ICP monitoring device [11,12] was used to measure the ICP with biceps flexion of a 10 lb weight for one minute. The ICP was 10 mmHg at baseline, over 90 mmHg at peak exercise, and 12 mmHg at one minute post exercise (Table 1, Figure 3). Given the high peak exercise pressure and bilateral nature of the symptoms, the patient was diagnosed with bilateral forearm CECS and offered open four-compartment fasciotomies after discussion regarding the potential benefits and risks.

**Figure 4:**
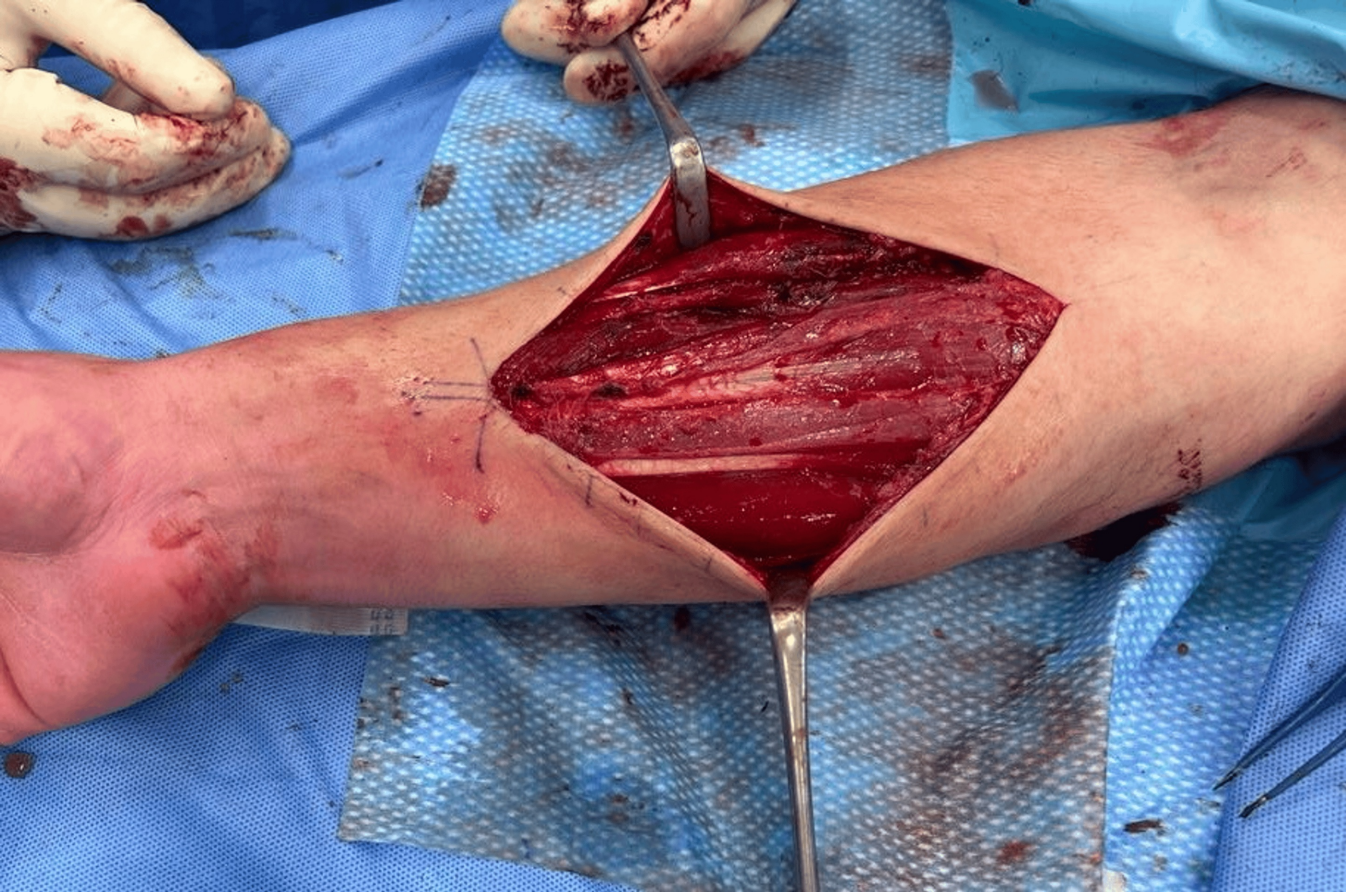
Release of the muscle bellies in the flexor compartment of the right forearm

**Figure 5:**
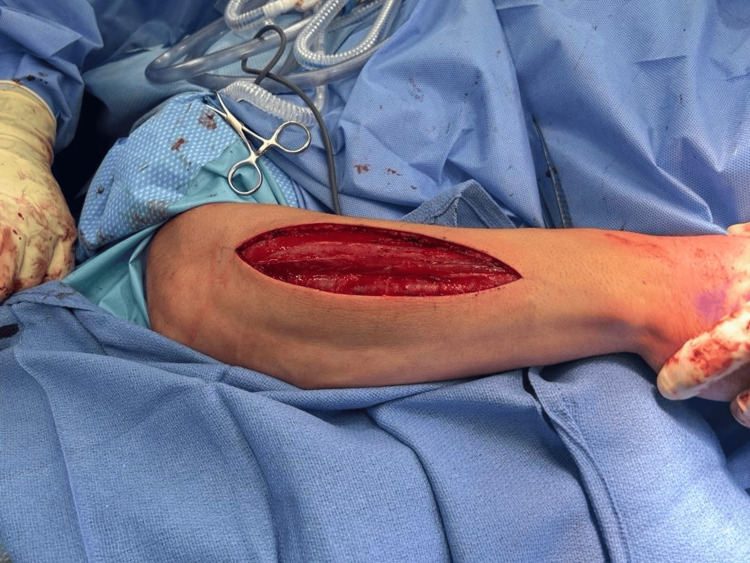
Release of the muscle bellies in the extensor compartment of the right forearm

The patient underwent bilateral fasciotomy of the superficial volar, deep volar, dorsal, and mobile wad compartments. A volar Henry approach was used for exposure to the volar and mobile wad compartments using a 12 cm incision. Decompression of each muscle belly was performed with notable bulging of the flexor digitorum superficialis (FDS) muscle with release of the overlying fascia (Figure 4). A 12 cm incision dorsal Thompson approach was used to access the extensor compartment. Individual muscle fasciae were released throughout the compartment (Figure 5). The same procedure was repeated on the contralateral side, with again a notable response to the release of the fascia overlying FDS. The surgical sites were irrigated, and the layers superficial to the fascia were closed with respect to anatomy. There were no surgical complications, and the patient had a favorable postoperative evolution. He returned to hockey six weeks after the procedure without any symptoms of CECS. At one-year follow-up, the patient had resumed competitive ice hockey and reported improved performance with no symptom recurrence. On examination, the wounds were well healed, and neurovascular examinations were normal.

## Discussion

We present a case of CECS involving all four limbs occurring in an elite male ice hockey athlete. CECS is classically seen in running athletes and marching military members when the lower limbs are involved and in motorcyclists, rowers, and kayakers when the upper limbs are involved. To our knowledge, this is the first report of a case of CECS in all four limbs of an individual and in an ice hockey athlete.

CECS is thought to be caused by decreased blood flow secondary to transiently elevated compartment pressures during exercise. Muscle hypertrophy, impediment to venous return, and noncompliant fascia are thought to play a role [7]. In the present case, repeated activation of the gastrocnemius-soleus complex upon pushing off during ice-skating was the likely trigger in the lower limbs. In the forearms, the trigger is less clear but appears to involve prolonged gripping of the hockey stick. The history was highly suggestive of CECS, and continuous intracompartmental measurements were helpful in confirming the diagnosis. At present, the diagnostic criteria for CECS of the lower limbs are better described, while there remains little consensus on pressure cut-offs for the upper limbs [6,7]. Our patient’s ICP measurements met all three Pedowitz criteria [5], with a notably elevated peak-exercise pressure of 108 mmHg. With these findings, we question whether peak-exercise ICP or continuous ICP monitoring can provide improved granularity in diagnosing CECS. Other diagnostic modalities, including MRI and near-infrared spectroscopy, have also been proposed [10,13].

Treatment of CECS includes non-operative options such as botulinum toxin injections, alteration of foot-strike pattern in runners, or cessation of the triggering activities [7,14]. However, patients are generally reluctant to give up the sport or hobby generating the symptoms [15]. In the case presented, fasciotomy of the four compartments of the lower limbs was effective in achieving complete resolution of symptoms without complication. In the forearms, extensive fasciotomy of the individual muscle bellies achieved the same result. Of note, a variety of surgical treatment modalities are described in the literature, including wide open fasciotomy, minimal incision fasciotomy, and endoscopic compartment release [15-17]. Ultimately, randomized controlled trials are necessary to determine superiority among these options.

CECS is a rare but important diagnosis for clinicians to consider in their differential diagnosis; 95% of CECS involve the lower limbs, with the remaining 5% distributed between the forearms, thighs, hands, and feet [18]. Thus, CECS of the forearms is an even rarer diagnosis, with limited literature available. The epidemiology of forearm CECS is not as well defined, but it occurs predominantly in athletes and individuals engaging in repetitive upper extremity activities, such as motorcyclists, rowers, and weightlifters [6]. Interestingly, it is also known to occur in musicians such as violinists and pianists. Considering that our patient suffered symptoms of CECS occasionally while driving, it is important to consider the diagnosis of CECS in patients who are not athletes with relevant symptoms. Of the four forearm compartments, the superficial volar compartment is most commonly involved, and approximately one-third of patients have bilateral involvement [19]. Our patient likely had primary involvement of the superficial volar compartment, given the bulging of the FDS muscle belly with release of the overlying fascia intraoperatively. 

Given the rarity of forearm CECS, there is no consensus on ICP cutoffs during exercise for diagnosis [6]. However, it has been reported that an ICP greater than 30 mmHg in any compartment of the forearm generally supports a diagnosis of CECS [20]. In the present case, the criteria outlined by Pedowitz et al. [5] for the lower limbs were used as a reference. In addressing these challenges, the utilization of a continuous ICP monitor allowed for the assessment of the trend of the ICP over time in correspondence to the timing of the exercise test, which provided additional information to aid in the diagnostic process. Further, this method theoretically allows for the identification of falsely elevated compartment pressures at initial insertion of the device due to muscle spasm, minimizing the risk of overdiagnosis and unnecessary risks associated with surgical intervention. With the use of a continuous ICP monitor, it is worth considering confounding factors or limitations to the reliability of readings; for example, the effect of external factors such as the psychological stress of the patient or the variability in effort provided by different patients in the same exercise test. Presently, there is scarce, if any, literature pertaining to this topic. With additional research utilizing this continuous ICP monitor, it may be possible to define criteria for the diagnosis of CECS specific to the forearms. Ultimately, this case underscores the need for further research and standardized guidelines in diagnosing and managing CECS of the forearms, as the condition's unique presentation and less-developed diagnostic criteria continue to pose challenges for clinicians.

## Conclusions

The present case study demonstrates that CECS can occur in ice hockey athletes, a sport that is not traditionally associated with this rare condition. It should therefore be included in the differential diagnosis when a suggestive history is provided, even when the associated activity is not a classic cause. In addition, CECS can occur in the lower limbs and forearms. Continuous ICP monitoring during physical activity is a fast and efficient tool to help clinicians in the diagnosis of CECS. However, larger studies will be required to establish the diagnostic criteria in the forearms. Open fasciotomy can be effective to manage cases of CECS refractory to conservative management in the lower and upper extremities.
